# Recurrent Thymic Carcinoma Treated With Median Sternotomy, Innominate Vein Replacement for Superior Vena Cava, and Iodide Implantation: A Case Report and Review of the Literature

**DOI:** 10.1002/cnr2.70089

**Published:** 2025-01-24

**Authors:** Zhong‐zheng Chen, Wen‐dong Qu, Xing‐shu Zhang, Yong‐xiang Song

**Affiliations:** ^1^ Department of Thoracic Surgery Affiliated Hospital of Zunyi Medical College Zunyi People's Republic of China

**Keywords:** chemotherapy, immunotherapy, neuroendocrine tumors of the thymus (NETT), superior vena cava, surgical resection, thymic carcinoma

## Abstract

**Background:**

Neuroendocrine tumors of the thymus (NETT) are rare and malignant tumors that arise in the anterior mediastinum. These tumors can exhibit aggressive behavior and may involve surrounding critical structures, such as the superior vena cava. This case contributes to the literature by presenting a recurrent thymic carcinoma with invasion of major blood vessels, including the superior vena cava, and the complexities involved in its surgical management.

**Case:**

A 51‐year‐old male with no significant medical history presented with eyelid edema and a mediastinal mass. Diagnostic imaging, including positron emission tomography/computed tomography (PET/CT), revealed a malignant anterior mediastinal mass with possible metastasis. The patient underwent thoracoscopic resection of the tumor and wedge resection of the left upper lobe of the lung. Postoperative pathology confirmed a neuroendocrine carcinoma (G3), staged as Masaoka IVa. Despite aggressive surgery, the patient developed recurrent metastasis involving mediastinal lymph nodes and the superior vena cava. The patient underwent complex surgery involving vascular replacement, pericardial resection, and superior vena cava reconstruction, followed by adjuvant chemotherapy, radiotherapy, and immunotherapy.

**Conclusion:**

This case highlights the challenges of managing advanced NETT, particularly with invasion of major vascular structures. It emphasizes the importance of early diagnosis, complete surgical resection, and tailored adjuvant therapies, including chemotherapy, radiotherapy, and immunotherapy, to improve survival outcomes. The lessons learned from this case contribute to refining treatment strategies for similar cases, advocating for aggressive surgical approaches and the potential benefit of novel therapeutic agents in the management of advanced NETT.

**Abbreviations**
AJCC: American Joint Committee on Cancer
CT: computed tomography
JLCS: Japanese Lung Cancer Societies
MDT: multidisciplinary treatment
NCCN: National Comprehensive Cancer Network
NEC: neuroendocrine carcinoma
NETT: neuroendocrine tumors of the thymus
OS: overall survival
PET/CT: positron emission tomography/computed tomography
PFS: progression‐free survival
VATS: video‐assisted thoracoscopic surgery
WHO: World Health Organization

## 1 Introduction

Neuroendocrine tumors of the thymus (NETT) are rare malignant tumors with neuroendocrine functions, located in the anterior mediastinum thymic region. The origin of these tumors is not yet clear. According to the 2004 World Health Organization (WHO) classification, NETT is an epithelial tumor that is either entirely or predominantly composed of neuroendocrine cells. Therefore, the presence of a few neuroendocrine differentiation clusters in thymic carcinoma does not belong to NETT. In the 2015 WHO classification, NETT is divided into low‐grade typical carcinoid, intermediate‐grade atypical carcinoid, high‐grade large cell neuroendocrine carcinoma (NEC), and small cell NEC [1]. Thymic carcinoma is highly malignant and can invade surrounding fat, pericardium, pleura, major blood vessels, and lungs at an early stage [2]. According to the literature, 40.0%–60.0% of thymic carcinoma patients are already in stage Masaoka Ill at the time of diagnosis. For such locally advanced patients, there is currently no unified treatment strategy [3, 4]. However, several studies advocate for complete resection of the lesion and invaded surrounding tissues and organs to improve patient prognosis [3, 4, 5, 6] A recent study by Hsu et al. emphasized that surgical intervention remains the cornerstone of treatment, particularly for invasive cases, while also highlights the variability in patient outcomes and the need for tailored postoperative management.

This case contributes to filling existing gaps in the literature by presenting a detailed account of a unique presentation and management of a recurrent thymic carcinoma. It offers insights into the complexities of treatment approaches, particularly for cases involving invasion of critical structures such as the superior vena cava, thus aiming to enhance understanding and improve management strategies for similar cases in clinical practice.

The presented patient was treated in the departments of thoracic surgery, Affiliated Hospital of Zunyi Medical College, China. A written informed consent was obtained from the patient for publication of this case report. The patient received detailed information about the therapeutic strategy and operative procedure.

## 2 Case Presentation

**FIGURE 1:**
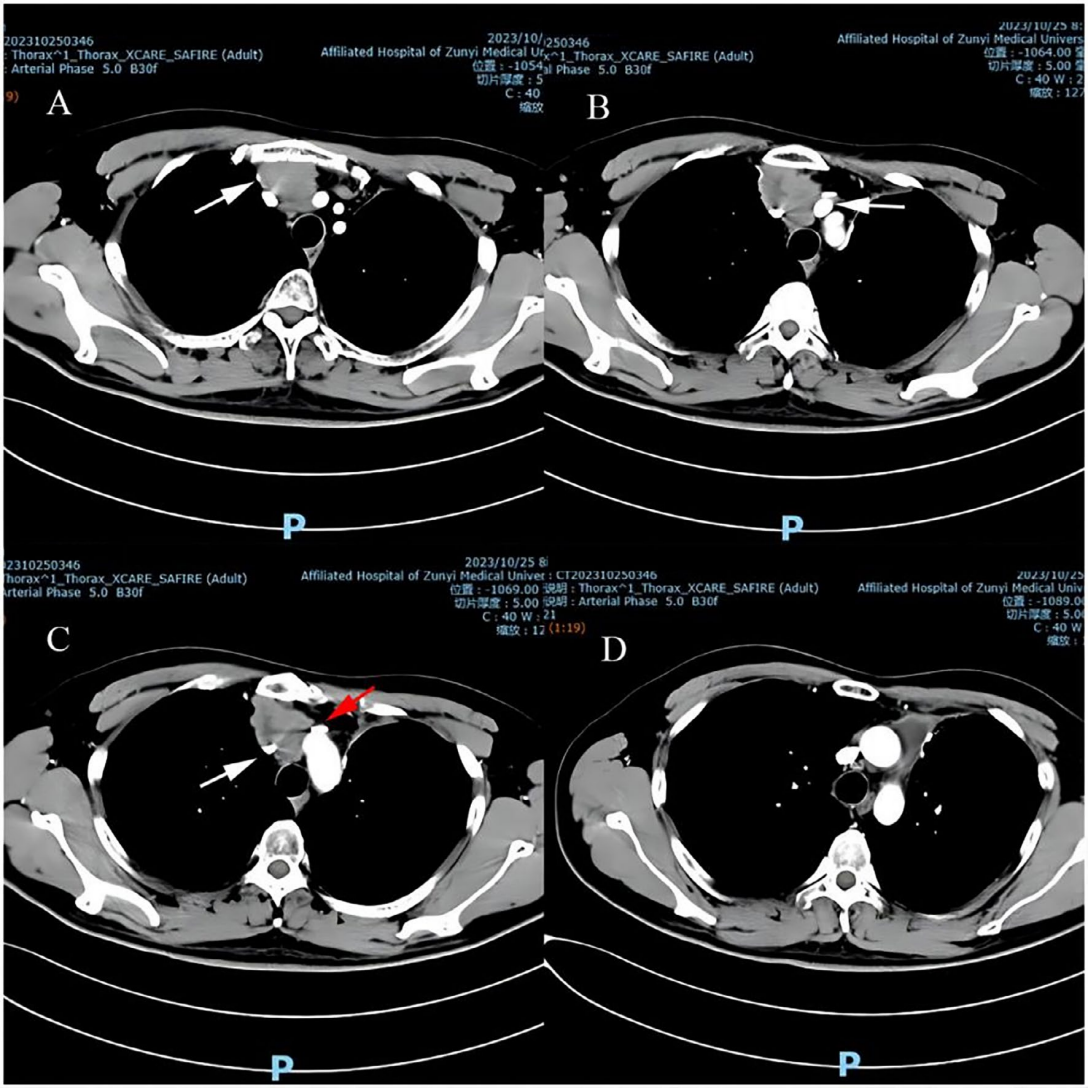
Time‐lapse computer tomography (CT) imaging of the chest of the patient with thymoma. (A) White arrow indicates right brachiocephalic vein. (B) White arrow indicates brachiocephalic trunk. (C) White arrow indicates superior vena cava, red arrow indicates left brachiocephalic vein. (D) Continuation of the C layer and can be left unmarked and uninterpreted.

A 51‐year‐old male with no previous medical history, no smoking history, and no history of chronic lung disease. The patient presented to The General Hospital of the People's Liberation Army on November 30, 2021, with the chief complaint of “Eyelid edema in the morning for 1 month” Chest three‐dimensional computed tomography (CT) revealed a mass shadow in the anterior superior mediastinum. Further positron emission tomography/computed tomography (PET/CT) examination at Zunyi Medical University Affiliated Hospital on November 23, 2021, indicated an anterior mediastinal mass with increased metabolism, suggesting malignant lesions, with enlargement of mediastinal lymph nodes 3a and 6, indicating possible metastasis. After excluding relevant surgical contraindications, the patient underwent “thoracoscopic resection of anterior mediastinal tumor and wedge resection of the left upper lobe of the lung” on December 2, 2021. Postoperative pathology revealed (anterior mediastinum) NEC, G3 with necrosis, tumor invasion into the lung parenchyma, and > 20 mitotic figures/10 high‐power fields. Lymph nodes in the hilum showed no metastatic carcinoma (0/1). Immunohistochemistry results were positive for CK, Syn, CgA, CD56, and Ki‐67 (40%) and negative for S‐100. Pathological diagnosis: Masaoka stage IVa. Postoperative follow‐up chest CT showed partial loss of the left upper lobe of the lung, with a small amount of pneumonia and localized lung consolidation. Postoperative diagnosis: T3NOMO stage IIIA, based on the 8th edition of the American Joint Committee on Cancer (AJCC) staging system. The patient received six cycles of chemotherapy postoperatively (specific chemotherapy regimen: Carboplatin + etoposide, palbociclib injection 200 mg intravenous infusion on Day 0, carboplatin injection [Qilu] 600 mg intravenous infusion on Day 1, etoposide injection 180 mg intravenous infusion from Day 1 to Day 3). The patient also underwent one course of radiotherapy (specific radiotherapy regimen: Total irradiation dose of the lesion was 50 Gy, with a single dose of 2 Gy, administered twice daily, five times a week [Monday–Friday], for a total of 25 session‐s [fx]). Subsequently, the patient received regular immunotherapy at our hospital (specific regimen: Palbociclib 200 mg intravenous infusion). Following that, regular chest CT follow‐ups were conducted. On October 25, 2023, the patient underwent a chest CT review, which showed enlarged lymph nodes in the anterior superior mediastinum, indicating multiple lymph node metastases and narrowing of the adjacent superior vena cava. There was partial loss of the left upper lobe of the lung, with a small amount of chronic inflammation. There was a small amount of fluid accumulation in the anterior mediastinum and thickening of the left local pleura. Compared to the previous chest CT, there was a significant increase in the size of the anterior superior mediastinal lymph nodes. (Figure 1) Consideration of recurrent metastasis of thymic carcinoma. Clinical diagnosis of the patient: Stage IVB, T3N2M1a. Preoperative comprehensive examinations: Abdominal CT, abdominal and pelvic ultrasound, and brain CT scan showed no evidence of other tumorous lesions. Serum tumor markers CEA, CA19‐9, AFP, and β‐hCG were within the normal range.

### 2.1 Operation

**FIGURE 2:**
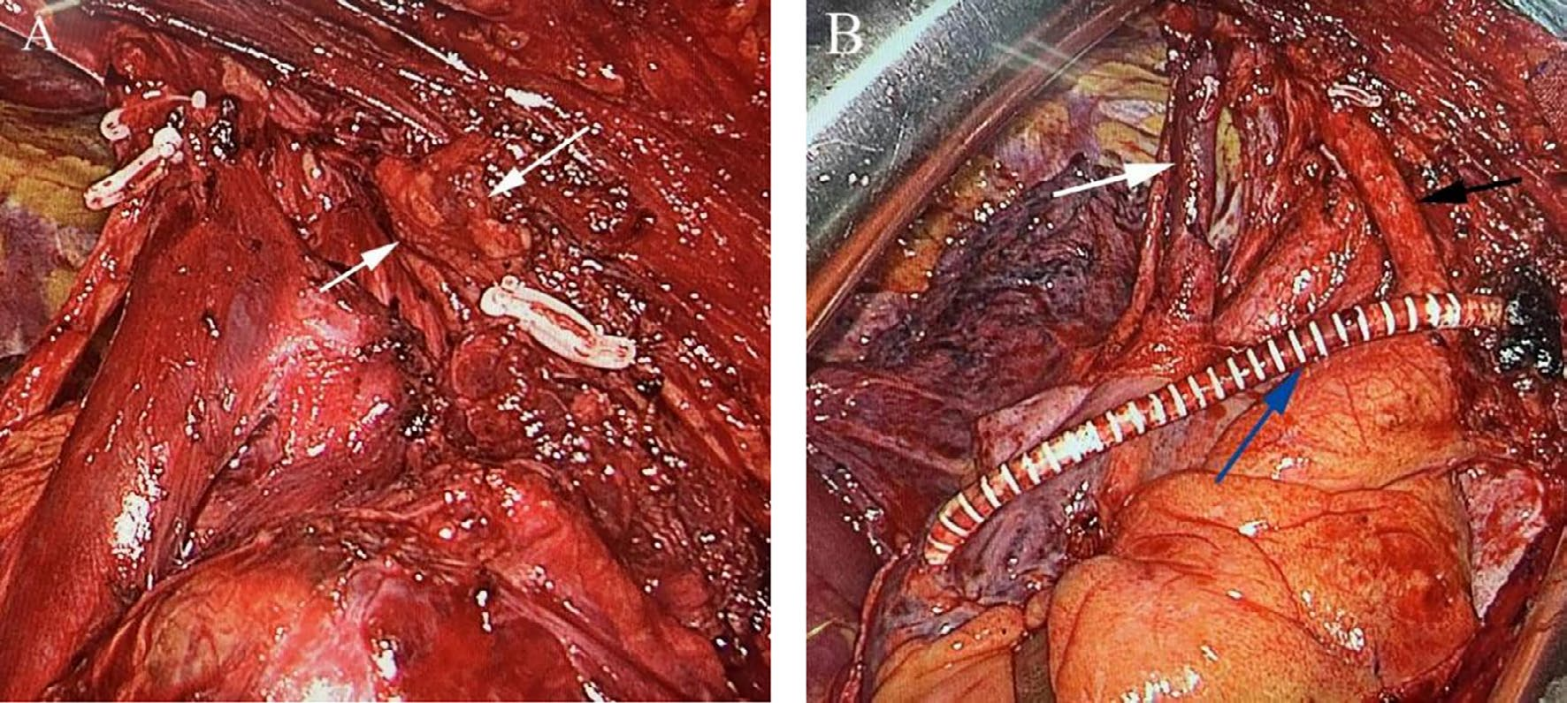
Intraoperative imaging (A) the white icon indicates the area where the tumor invades the brachiocephalic vein. (B) The white icon represents the reconstructed superior vena cava, the black icon represents the brachiocephalic trunk, and the blue icon represents the artificial blood vessel graft from the brachiocephalic vein to the right atrium.

The surgical approach was determined based on the patient's medical history and imaging findings, as discussed in a multidisciplinary treatment (MDT) consultation. Considering the patient's history and imaging manifestations, recurrent thymic carcinoma was suspected, with involvement of the pericardium, bilateral brachiocephalic veins, superior vena cava, and brachiocephalic trunk. The surgery involved vascular replacement, extensive pericardial resection, left brachiocephalic vein‐right atrium bypass, superior vena cava reconstruction, and iodine‐125 radioactive seed implantation for close‐range treatment. Routine anesthesia intubation and sterile draping were performed, and a mid‐sternal incision was made. Intraoperatively, the tumor was observed in the anterior upper mediastinum, slightly to the right, measuring 4.1 × 4.0 × 4.2 cm, solid in consistency, firmly adherent to the surface of the pericardium, and located anterior to the aortic arch. The upper pole of the tumor was positioned between the right brachiocephalic vein and the brachiocephalic trunk, with its right margin closely adjacent to the superior vena cava and its left margin invading the left brachiocephalic vein. The tumor completely encased the initial segment of the brachiocephalic vein, extending posteriorly along the brachiocephalic trunk to the descending aorta. The tumor extended inferiorly to the level of the lower border of the aortic arch, involving the pericardium in this region. The tumor capsule appeared uneven, suggesting possible invasion of the pleura. The tumor also invaded the base of the heart (measuring approximately 5.0 × 4.8 × 5.2 cm). The right pleura was opened, and upon entering the right pleural cavity, it was observed that the right lung was not affected. The left pleura showed localized adhesions and was not entered. During the surgery, the visible primary lesion, the surrounding involved tissues, the invaded pleura, pericardium, superior vena cava, and left brachiocephalic vein were all resected. The artificial blood vessel was anastomosed end‐to‐end with the left brachiocephalic vein and the right atrial end. The left brachiocephalic vein was excised, and the artificial blood vessel was grafted (Figure 2). The tumor was further dissected toward the upper right region, revealing the bilateral superior thymic veins, which were completely freed and resected, along with the branches of multiple thymic veins. The proximal ends were occluded with vascular clips, and the distal ends were divided using an ultrasonic scalpel. The thymus, along with the surrounding tissue, was completely excised. The tumor was then pulled toward the upper right region, ensuring full mobilization of the right phrenic nerve, vagus nerve, and trachea. The relationship between the tumor and the right brachiocephalic vein from its origin to the mid‐segment of the superior vena cava was found to be tight. After careful dissection using scissors, the tumor involving the base of the superior vena cava was partially occluded with a side‐wall vascular clamp, and the tumor and a portion of the vascular wall of the superior vena cava were resected. The tumor tissue was removed, and 5‐0 suture was used for vascular closure. The side wall of the superior vena cava was continuously sutured using the clamps, and superior vena cava reconstruction was performed. lodine‐125 radioactive seeds were implanted in the area of the tumor in the anterior mediastinum. Hemostasis was achieved, and two drainage tubes were placed in the right pleural cavity and the anterior mediastinal region through the bilateral rib arches. The sternum was closed with steel wire sutures, and the chest was closed layer by layer. The operation was completed. The patient received postoperative care, including antibiotic therapy, analgesia, heart rate management, and anticoagulation. The anticoagulation protocol involved a daily subcutaneous injection of 4000 IU enoxaparin sodium, initiated on postoperative Day 1 and continued for 5 days. The follow‐up strategy includes a chest X‐ray at 1 week postoperatively to monitor for pneumothorax and pleural effusion; a chest CT scan with 3D reconstruction at 1 month to evaluate the reconstructed vessels and surgical site; and a repeat CT scan at 6 months to detect any signs of tumor recurrence or metastasis. Of particular note, the patient's chest CT at 1 month revealed significant right‐sided pleural effusion, approximately 60 mm in thickness. The patient was subsequently rehospitalized and underwent a right pleural closed drainage procedure after excluding surgical contraindications, with approximately 850 mL of pale‐yellow pleural fluid drained. The patient was discharged 1 week later in full recovery.

### 2.2 Histopathological and Immunohistochemical

**FIGURE 3:**
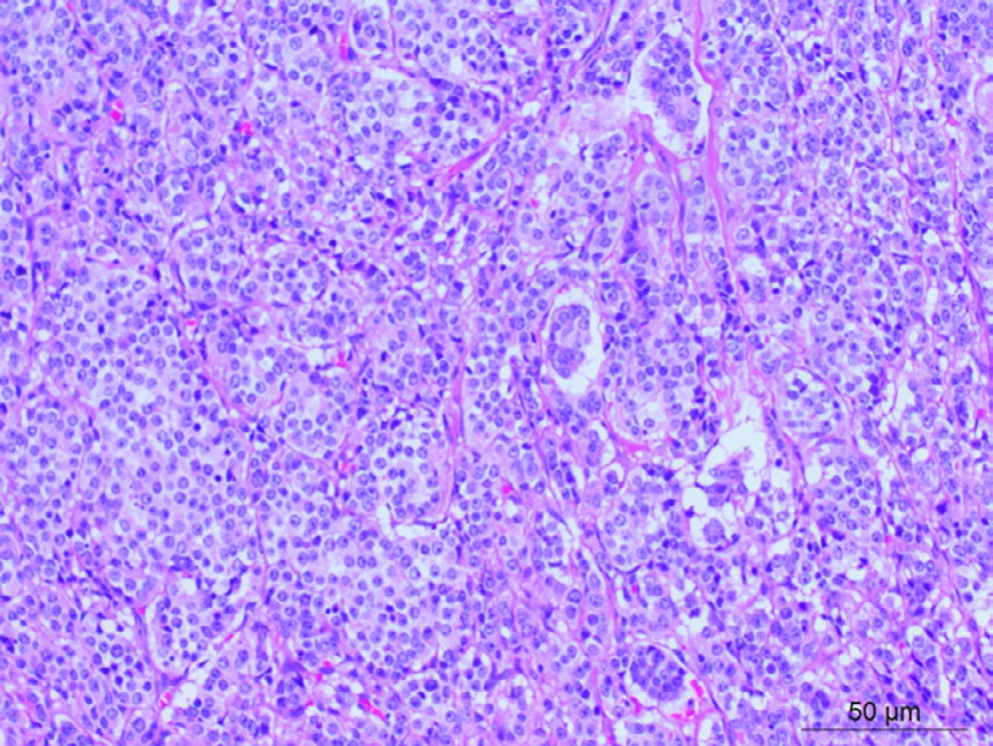
H&E stain shows the histology of the malignant thymoma. This was evidenced by the presence of a mixture of plump epithelial cells with both vesicular nuclei and distinct nucleoli and small lymphocytes (magnification = ×200).

**FIGURE 4:**
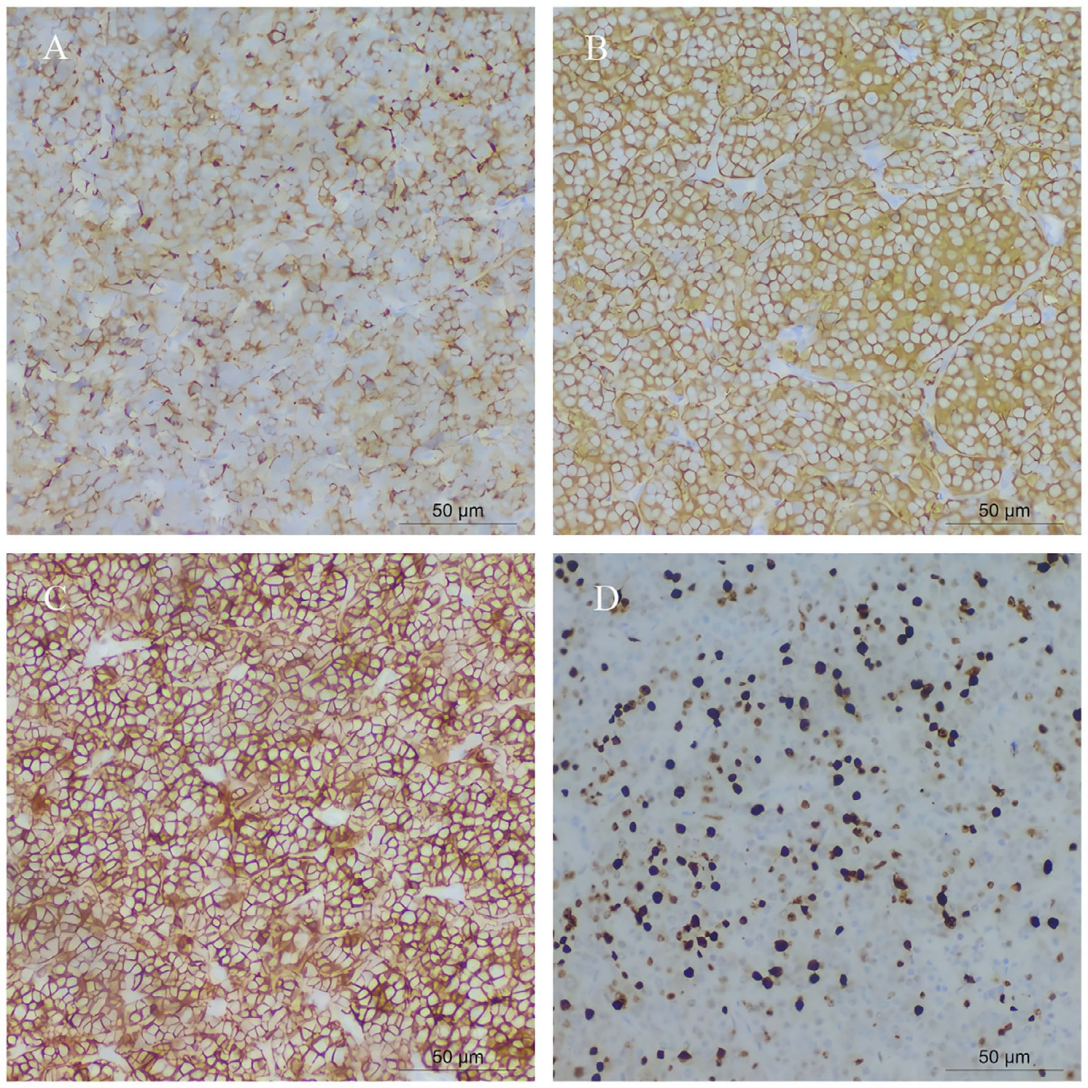
Shows immunohistochemical staining specific to the thymic neuroendocrine tumor. (A) (CgA) demonstrates strong cytoplasmic positivity in tumor cells, indicating the presence of neuroendocrine granules (magnification = ×200). (B) (Syn) reveals diffuse positivity in the cytoplasm of the tumor cells, further confirming the neuroendocrine differentiation (magnification = ×200). (C) (CD56) shows membranous positivity, highlighting the neuroendocrine nature of the tumor cells (magnification = ×200). (D) (Ki‐67) illustrates a proliferative index with approximately 40% of tumor cells showing nuclear staining, indicating the proliferative activity of the tumor (magnification = ×200).

Postoperative pathology confirmed a complete tumor resection with negative margins (R0), Postoperative pathological findings: (anterior mediastinum) atypical carcinoid tumor (approximately 5 mitoses/10 high‐power fields, hot spot area Ki‐67 proliferation index approximately 40%+); additional specimens from (anterior mediastinum) adipose tissue and (left margin) showed no evidence of tumor tissue. Lymph nodes around the mass (3/3), lymph nodes from the 2nd and 4th groups (2/2), and cervical lymph node (1/1) showed tumor metastasis, while mediastinal lymph nodes (0/2) showed no tumor metastasis (Figures 3 and 4). Immunohistochemical findings: Tumor cells positive for CK (+++), Vimentin (scattered +), ATRX (+++), CD56 (+++), Syn (+++), CgA (++), P53 (scattered weak to moderate intensity +), SSTR2 (occasional +), Ki‐67 (approximately 40%+ in hot spot area), C7 (−).

## 3 Discussion

The most common type of primary thymic carcinoma is squamous cell carcinoma, with some rare subtypes including basaloid carcinoma, mucoepidermoid carcinoma, lymphoepithelioma‐like carcinoma, clear cell carcinoma, sarcomatoid carcinoma, and neuroendocrine tumors [7]. NETT is extremely rare and was first reported by Rosai et al. [8] in 197, including eight patients.

Currently, only over 500 cases have been reported domestically and internationally [9, 10, 11, 12, 13]. In the 2015 WHO classification of thymic tumors, thymic neuroendocrine tumors are divided into five subtypes: typical carcinoid, atypical carcinoid, large cell NEC, thymic carcinoma combined with large cell NEC, small cell carcinoma, and thymic carcinoma combined with small cell carcinoma. This study summarizes over 500 cases of thymic neuroendocrine tumors reported in the literature. The average age of onset for patients is 57 years, with a male‐to‐female ratio of 2.5:1. Male patients are more common, and the average diameter of the tumor is 7.3 cm. Some patients experience symptoms such as chest tightness, chest pain, shortness of breath, and respiratory distress, while others are asymptomatic, and the tumor is detected during physical examination as a mediastinal mass. This case involves a 51‐year‐old male with a tumor diameter of 4.1 × 4.0 × 4.2 cm. A mediastinal mass was detected during a physical examination, consistent with the literature reports. In approximately one‐third of patients, NETT presents as an asymptomatic mass in the anterior mediastinum. It rarely occurs in the middle or posterior mediastinum. NETT can exhibit local invasiveness, leading to displacement or compression of the chest structures. The reported median size is approximately 8 cm. Patients may experience symptoms and signs such as cough, chest pain, shortness of breath, or superior vena cava obstruction. Additionally, at the time of diagnosis, up to 62% of patients may have mediastinal lymph node metastasis, while distant metastasis occurs in 20%–30% of patients, most commonly affecting the bones, lungs, and liver [14].

In 1987, Dartevelle first reported a series of 13 cases utilizing synthetic grafts to replace the superior vena cava invaded by lung and mediastinal tumors, thereby pioneering the exploration of vascular reconstruction feasibility [15]. In 2017, Sun and colleagues conducted a retrospective analysis of invasive thymic tumors, emphasizing the safety and efficacy of vascular reconstruction [16]. In 2004, Park and collaborators summarized surgical strategies for thoracic malignancies involving the heart and major vessels, detailing surgical approaches and the extent of resection [17]. In 2022, Lampridis and co‐authors reported a case of extensive invasive thymoma encroaching upon the superior vena cava and left brachiocephalic vein, reaffirming the safety and efficacy of surgical intervention [18]. Surgery is the primary treatment method for NETT, and complete resection is crucial. Literature indicates that RO resection improves patient survival [19, 20]. Crona et al. demonstrated 100% 5 and 10‐year survival rates in three patients who underwent RO resection, whereas mixed group patients had a 5‐year survival rate of 76% and a 10‐year survival rate of 32%. Sullivan's study showed an average survival of 109 months for surgically resected patients compared to 38 months for patients who underwent debulking procedures only. Although complete resection is ideal, in certain patients, even partial resection may offer better survival outcomes compared to no surgery at all. In cases of disease recurrence, surgery is only necessary when the disease is considered resectable. Currently, median sternotomy has been the standard approach, but video‐assisted thoracoscopic surgery (VATS) through intercostal incisions is becoming increasingly common. The guidelines from the European and Japanese Lung Cancer Societies (JLCS) for thymic tumors, including thymic carcinoma and NETT, allow experienced surgeons to use VATS for stage I and Il tumors [21, 22]. Further technological advancements, such as VATS using subxiphoid approach instead of intercostal incisions, and the use of robotic surgery, have demonstrated the potential benefits of thymectomy. Despite these advancements, the choice of surgical approach still depends on factors such as tumor size, extent of local structural damage, available equipment, and surgeon preferences and experience. The principle of achieving clear resection margins with minimal morbidity remains crucial.

The patient was initially diagnosed with stage IIIB mediastinal tumor. The first surgery was performed using VATS to remove the mediastinal mass. After surgery, the patient underwent six cycles of chemotherapy and one course of radiation therapy. Additionally, the patient received pembrolizumab as adjuvant immunotherapy. We have two concerns regarding this treatment plan: (1) For locally advanced mediastinal tumors that have invaded lung tissue, can VATS completely remove the lesion and achieve R0 resection? (2) There is controversy between the National Comprehensive Cancer Network (NCCN) and domestic guidelines regarding the recommendation of immunotherapy for adjuvant treatment of thymoma [23, 24, 25, 26]. Currently, clinical trials both domestically and internationally have primarily focused on using immunotherapy after platinum‐based chemotherapy failure [27, 28]. Whether the combination of chemotherapy and immunotherapy can prolong the progression‐free survival (PFS) and overall survival (OS) of patients with thymic cancer is not yet known. After the patient's tumor recurrence was detected in our hospital, it was discussed in an MDT meeting. Considering the short disease‐free survival after the previous surgery and adjuvant radiation therapy, preoperative imaging results showed tumor invasion surrounding the pericardium, innominate vein, and superior vena cava. Intraoperatively, the tumor was found to be closely adhering to the right margin of the superior vena cava, and significant bleeding occurred during the dissection. The possibility of artificial vessel replacement in the superior vena cava was evaluated to ensure unobstructed cerebral venous return during the replacement surgery. Therefore, a left innominate vein‐right atrium artificial vessel bypass was performed as a preliminary procedure to reduce the risk of cerebral edema caused by impaired cerebral venous return. During the continued exploration, it was observed that the tumor pedicle involved less than one‐third of the wall of the superior vena cava, and it was evaluated as feasible to perform superior vena cava reconstruction, which could achieve complete tumor resection. After the completion of the reconstructive surgery, the area of superior vena cava reconstruction was significantly narrowed, but the establishment of the left innominate vein bypass effectively relieved the blood reflux resistance caused by the stenosis in that area. Iodine particle placement was performed after tumor resection to serve as a preventive radiation therapy and to ensure complete tumor dissection and reduce the risk of bleeding and implantation metastasis. In this case, the patient chose a median sternotomy for the procedure. However, this case report has several limitations: (1) The follow‐up duration is relatively short, only 9 months as of August 2024, limiting the assessment of long‐term disease‐free survival and OS. (2) The findings of this study may not be generalizable. (3) Potential biases among the researchers involved could affect the study's conclusions.

## 4 Conclusions

NETT is extremely rare. For locally advanced patients with involvement of the superior vena cava and surrounding blood vessels, aggressive surgical intervention should be pursued along with adjuvant therapies to improve patient prognosis. Currently, early diagnosis followed by complete surgical resection remains the best option for prolonging patient survival. The diversification of chemotherapy regimens and the development of novel agents in the treatment of NETTs, including kinase inhibitors, mTOR inhibitors, radiopharmaceutical therapy, and immunotherapy, may potentially improve the prognosis of patients with more advanced NETs.

## Author Contributions

**Zhong‐zheng Chen:** writing – original draft, writing – review and editing. **Wen‐dong Qu:** data curation. **Xing‐shu Zhang:** visualization. **Yong‐xiang Song:** writing – review and editing, methodology, writing – original draft.

## Ethics Statement

The authors have nothing to report.

## Consent

A written informed consent was obtained from the patient for publication of this case report and any accompanying images. A copy of the written consent is available for review.

## Conflicts of Interest

The authors declare no conflicts of interest.

## Data Availability

Data sharing not applicable to this article as no datasets were generated or analysed during the current study.

## References

[1] W. D. Travis , E. Brambilla , A. P. Burke , A. Marx , and A. G. Nicholson , “Introduction to the 2015 World Health Organization Classification of Tumors of the Lung, Pleura, Thymus, and Heart,” Journal of Thoracic Oncology 10, no. 9 (2015): 1240–1242. 26291007 10.1097/JTO.0000000000000663

[2] H. Kawasaki , N. Taira , T. Ichit , et al., “Weekly Chemotherapy With Cisplatin, Vincristine, Doxorubicin, and Etoposide Followed by Surgery for Thymic Carcinoma,” European Journal of Surgical Oncology 40, no. 9 (2014): 1151–1155. 24703656 10.1016/j.ejso.2014.03.006

[3] K. Kondo and Y. Monden , “Therapy for Thymic Epithelial Tumors: A Clinical Study of 1320 Patients From Japan,” Annals of Thoracic Surgery 76, no. 3 (2003): 878–884. 12963221 10.1016/s0003-4975(03)00555-1

[4] H. Fu , Z. T. Gu , W. T. Fang , et al., “Long‐Term Survival After Surgical Treatment of Thymic Carcinoma: A Retrospective Analysis From the Chinese Alliance for Research of Thymoma Database,” Annals of Surgical Oncology 23, no. 2 (2016): 619–625. 26474558 10.1245/s10434-015-4825-4

[5] Y. Zhang , D. Lin , B. Aramini , et al., “Thymoma and Thymic Carcinoma: Surgical Resection and Multidisciplinary Treatment,” Cancers 15, no. 7 (2023): 1953. 37046614 10.3390/cancers15071953 PMC10093507

[6] B. Weksler , R. Dhupar , V. Parikh , K. S. Nason , A. Pennathur , and P. F. Ferson , “Thymic Carcinoma: A Multivariate Analysis of Factors Predictive of Survival in 290 Patients,” Annals of Thoracic Surgery 95, no. 1 (2013): 299–303. 23141529 10.1016/j.athoracsur.2012.09.006

[7] C. A. Moran and S. Suster , “Neuroendocrine Carcinomas (Carcinoid Tumor) of the Thymus: A Clinicopathologic Analysis of 80 Cases,” American Journal of Clinical Pathology 114, no. 1 (2000): 100–110. 10884805 10.1309/3PDN-PMT5-EQTM-H0CD

[8] J. Rosai and E. Higa , “Mediastinal Endocrine Neoplasm, of Probable Thymic Origin, Related to Carcinoid Tumor. Clinicopathologic Study of 8 Cases,” Cancer 29 (1972): 1061–1074. 4111691 10.1002/1097-0142(197204)29:4<1061::aid-cncr2820290456>3.0.co;2-3

[9] C. H. Hsu , J. K. Chan , C. H. Yin , C. C. Lee , C. U. Chern , and C. I. Liao , “Trends in the Incidence of Thymoma, Thymic Carcinoma, and Thymic Neuroendocrine Tumor in the United States,” PLoS One 14 (2019): e0227197. 31891634 10.1371/journal.pone.0227197 PMC6938371

[10] P. Strobel , A. Zettl , K. Shilo , et al., “Tumor Genetics and Survival of Thymic Neuroendocrine Neoplasms: A Multi‐Institutional Clinicopathologic Study,” Genes, Chromosomes & Cancer 53 (2014): 738–749. 24764238 10.1002/gcc.22183

[11] C. T. Bakhos , A. C. Salami , L. R. Kaiser , R. V. Petrov , and A. E. Abbas , “Thymic Neuroendocrine Tumors and Thymic Carcinoma: Demographics, Treatment, and Survival,” Innovations 15 (2020): 468–474. 32938293 10.1177/1556984520949287

[12] W. K. De Jong , J. L. Blaauwgeers , M. Schaapveld , et al., “Thymic Epithelial Tumours: A Population‐Based Study of the Incidence, Diagnostic Procedures, and Therapy,” European Journal of Cancer 44 (2007): 123–130. 10.1016/j.ejca.2007.11.004 18068351

[13] J. Y. Tang , H. J. Gao , G. D. Shi , et al., “Development and Validation of a Nomogram Prognostic Model for Patients With Neuroendocrine Tumors of the Thymus,” Thoracic Cancer 11, no. 9 (2020): 2457–2464. 32656987 10.1111/1759-7714.13556 PMC7471026

[14] J. Lau , T. I. Cvasciuc , D. Simpson , et al., “Continuing Challenges of Primary Neuroendocrine Tumours of the Thymus: A Concise Review,” European Journal of Surgical Oncology 48 (2022): 2360–2368, 10.1016/j.ejso.2022.07.017. 35922282

[15] P. Dartevelle , A. Chapelier , M. Navajas , et al., “Replacement of the Superior Vena Cava With Polytetrafluoroethylene Grafts Combined With Resection of Mediastinal‐Pulmonary Malignant Tumors. Report of Thirteen Cases,” Journal of Thoracic and Cardiovascular Surgery 94, no. 3 (1987): 361–366. 3626598

[16] Y. Sun , C. Gu , J. Shi , et al., “Reconstruction of Mediastinal Vessels for Invasive Thymoma: A Retrospective Analysis of 25 Cases,” Journal of Thoracic Disease 9, no. 3 (2017): 725–733, 10.21037/jtd.2017.03.03. 28449480 PMC5394000

[17] B. J. Park , M. Bacchetta , M. S. Bains , et al., “Surgical Management of Thoracic Malignancies Invading the Heart or Great Vessels,” Annals of Thoracic Surgery 78, no. 3 (2004): 1024–1030, 10.1016/j.athoracsur.2004.02.043. 15337042

[18] S. Lampridis , R. Bilkhu , G. Lucchese , and A. Billè , “Complete Surgical Resection of a Giant Invasive Thymoma With Right Pneumonectomy and Graft Reconstruction of the Superior Vena Cava and Left Brachiocephalic Vein: A Case Report,” Case Reports in Surgery 28, no. 2022 (2022): 9604926, 10.1155/2022/9604926. PMC9722296 36479542

[19] J. Crona , P. Bjrklund , S. Welin , G. Kozlovacki , K. berg , and D. Granberg , “Treatment, Prognostic Markers, and Survival in Thymic Neuroendocrine Tumours. A Study From a Single Tertiary Referral Centre,” Lung Cancer 79 (2012): 289–293. 10.1016/j.lungcan.2012.12.001 23286964

[20] J. L. Sullivan and B. Weksler , “Neuroendocrine Tumors of the Thymus: Analysis of Factors Affecting Survival in 254 Patients,” Annals of Thoracic Surgery 103 (2016): 935–939. 27720367 10.1016/j.athoracsur.2016.07.050

[21] K. Yokoi , K. Kondo , K. Fujimoto , et al., “JLCS Medical Practice Guidelines for Thymic Tumors: Summary of Recommendations,” Japanese Journal of Clinical Oncology 47 (2017): 1119–1122. 29036455 10.1093/jjco/hyx138

[22] N. Girard , E. Ruffini , A. Marx , C. Faivre‐Finn , S. Peters , and ESMO Guidelines Committee , “Thymic Epithelial Tumours: ESMO Clinical Practice Guidelines for Diagnosis, Treatment and Follow‐Up,” Annals of Oncology 26 (2015): v40–v55. 26314779 10.1093/annonc/mdv277

[23] C. Xu , Y. Zhang , W. Wang , et al., “Chinese Expert Consensus on the Diagnosis and Treatment of Thymic Epithelial Tumors,” Thoracic Cancer 14, no. 12 (2023): 1102–1117. 36924056 10.1111/1759-7714.14847 PMC10125784

[24] V. Tateo , L. Manuzzi , A. De Giglio , et al., “Immunobiology of Thymic Epithelial Tumors: Implications for Immunotherapy With Immune Checkpoint Inhibitors,” International Journal of Molecular Sciences 21, no. 23 (2020): 9056. 33260538 10.3390/ijms21239056 PMC7730788

[25] G. Giaccone and C. Kim , “Durable Response in Patients With Thymic Carcinoma Treated With Pembrolizumab After Prolonged Follow‐Up,” Journal of Thoracic Oncology 16, no. 3 (2021): 483–485. 33248322 10.1016/j.jtho.2020.11.003

[26] E. Baudin , M. Caplin , R. Garcia‐Carbonero , et al., “Lung and Thymic Carcinoids: ESMO Clinical Practice Guidelines for Diagnosis, Treatment, and Follow‐Up,” Annals of Oncology 32, no. 4 (2021): 439–451. 33482246 10.1016/j.annonc.2021.01.003

[27] J. Cho , H. S. Kim , B. M. Ku , et al., “Pembrolizumab for Patients With Refractory or Relapsed Thymic Epithelial Tumor: An Open‐Label Phase II Trial,” Journal of Clinical Oncology 37, no. 24 (2019): 2162–2170. 29906252 10.1200/JCO.2017.77.3184

[28] G. Giaccone , C. Kim , J. Thompson , et al., “Pembrolizumab in Patients With Thymic Carcinoma: A Single‐Arm, Single‐Centre, Phase 2 Study,” Lancet Oncology 19, no. 3 (2018): 347–355. 29395863 10.1016/S1470-2045(18)30062-7 PMC10683856

